# Progression into sepsis: an individualized process varying by the interaction of comorbidities with the underlying infection

**DOI:** 10.1186/s12879-018-3156-z

**Published:** 2018-05-29

**Authors:** Dimitrios Sinapidis, Vassileios Kosmas, Vasileios Vittoros, Ioannis M. Koutelidakis, Aikaterini Pantazi, Aggelos Stefos, Konstantinos E. Katsaros, Karolina Akinosoglou, Magdalini Bristianou, Konstantinos Toutouzas, Michael Chrisofos, Evangelos J. Giamarellos-Bourboulis

**Affiliations:** 1 Department of Therapeutics, National and Kapodistrian University of Athens, Medical School, Athens, Greece; 2 1st Department of Internal Medicine, “G.Gennimatas” Athens General Hospital, Athens, Greece; 3 1st Department of Internal Medicine, Thriasio Elefsis General Hospital, Magoula, Greece; 4 2nd Department of Surgery, Aristotle University of Thessaloniki, Thessaloniki, Greece; 5 2nd Department of Internal Medicine, Thriasio Elefsis General Hospital, Magoula, Greece; 6 Department of Medicine and Research Laboratory of Internal Medicine, Larissa University Hospital, University of Thessaly, Medical School, Volos, Greece; 7 Department of Surgery, Nafplion General Hospital, Nafplio, Greece; 8 Department of Internal Medicine, University of Patras, Rion, Greece; 9 Department of Internal Medicine, Lamia General Hospital, Lamia, Greece; 10 1st Department of Propedeutic Surgery, National and Kapodistrian University of Athens, Medical School, Athens, Greece; 11 2nd Department of Urology, National and Kapodistrian University of Athens, Medical School, Athens, Greece; 12 4th Department of Internal Medicine, National and Kapodistrian University of Athens, Medical School, Athens, Greece; 13 4th Department of Internal Medicine, ATTIKON University Hospital, 1 Rimini Street, 12462 Athens, Greece

**Keywords:** Infection, Sepsis, Comorbidities, Mortality, Intrabdominal

## Abstract

**Background:**

Development of sepsis is a process with significant variation among individuals. The precise elements of this variation need to be defined. This study was designed to define the way in which comorbidities contribute to sepsis development.

**Methods:**

Three thousand five hundred nine patients with acute pyelonephritis (AP), community-acquired pneumonia (CAP), intraabdominal infections (IAI) or primary bacteremia (BSI) and at least two signs of the systemic inflammatory response syndrome were analyzed. The study primary endpoint was to define how comorbidities as expressed in the Charlson’s comorbidity index (CCI) and the underlying type of infection contribute to development of organ dysfunction. The precise comorbidities that mediate sepsis development and risk for death among 18 comorbidities recorded were the secondary study endpoints.

**Results:**

CCI more than 2 had an odds ratio of 5.67 for sepsis progression in patients with IAI between significantly higher than AP and BSI. Forward logistic regression analysis indicated seven comorbidities that determine transition into sepsis in patients with AP, four comorbidities in CAP, six comorbidities in IAI and one in BSI. The odds ratio both for progression to sepsis and death with one comorbidity or with two and more comorbidities was greater than in the absence of comorbidities.

**Conclusions:**

The study described how different kinds of infection vary in the degree to which they lead to sepsis. The number of comorbidities that enhances the risk of sepsis and death varies depending on the underlying infections.

**Electronic supplementary material:**

The online version of this article (10.1186/s12879-018-3156-z) contains supplementary material, which is available to authorized users.

## Background

Despite progress in our understanding of the mechanism of pathogenesis, sepsis remains a leading cause of death. The Sepsis-3 expert committee developed diagnostic criteria for sepsis in which co-morbidities played a considerable role. According to their analysis, clinical signs prognostic of the added risk for death to the risk coming from comorbidities were used to develop the diagnostic criteria for sepsis [1]. The Charlson’s co-morbidity index (CCI) was applied by the Sepsis-3 expert panel as an expression of the co-morbidities [2].

Since 2006, the Hellenic sepsis study Group (HSSG) is collectively collecting clinical data for patients with infections presenting with at least two signs of the systemic inflammatory response syndrome (SIRS). Results from these studies on the traits of the innate and of the adaptive immune activation as well as on genotyping characteristics indicated that progression to organ dysfunction varied greatly among individuals and it was dependent on the type of infection [3, 4].

We have recently re-classified all the patients in our database into non-sepsis and sepsis according to the new Sepsis-3 definitions [5]. We asked the question if co-morbidities of patients admitted in the emergency department (ED) influence the development of organ dysfunction and whether this depends on the underlying infection. We tried to identify how each of the individual co-morbidities and how their constellation, expressed by the CCI, impacts on the development of organ failure and final outcome.

## Methods

### Study design

This is the analysis of the prospective collection of clinical information for patients admitted with at least two signs of SIRS at the ED of 38 hospitals in Greece from January 2007 until September 2016. The study protocol was approved by the Ethics Committees of the participating hospitals. Written informed consent was provided by the patients or by a legal representative in case of patients unable to consent. The study design and study endpoints were defined before the start of the study.

Inclusion criteria were: a) age equal to or more than 18 years; b) both genders; c) written informed consent; d) presence of at least two signs of SIRS as defined elsewhere [6]; and e) acute pyelonephritis (AP), community-acquired pneumonia (CAP), intraabdominal infections (IAI) and primary bacteremia (BSI) as the cause of SIRS. These infections were defined according to internationally accepted criteria [7–9]. Exclusion criteria were: a) age below 18 years; b) deny to consent; c) neutropenia defined as an absolute neutrophil count lower than 1000/mm^3^ for reason other than SIRS; and d) any metastatic solid tumor malignancy.

For all patients the following information was recorded: demographics, sequential organ failure assessment (SOFA) score, acute physiology and chronic health evaluation (APACHE) II score, CCI, co-morbidities and 28-day outcome. Eighteen comorbidities were recorded: type 2 diabetes mellitus, chronic heart failure, chronic obstructive pulmonary disorder (COPD), chronic renal disease, solid tumor malignancy, any hematological malignancy, chronic intake of corticosteroids, coronary heart disease, vascular hypertension, atrial fibrillation, dyslipidemia, obesity, history of stroke, dementia, nephrolithiasis, gallstones, liver cirrhosis and depression based on each patient medical history.

### Study endpoints

The study primary endpoint was to define if CCI interacts additively with the underlying type of infection for the development of organ dysfunction. At the original study protocol, organ dysfunctions were defined by the 2001 definitions. After the publication of the new Sepsis-3 definitions, it was decided to re-classify all patients in the database as non-sepsis and sepsis based on total SOFA score equal to or more than 2 [5].

The study secondary endpoints were: a) the precise comorbidities that influence development of sepsis within the subgroups of patients with a specific infection; b) the influence of the number of comorbidities for the development of sepsis within the subgroups of patients with a specific infection; c) the precise comorbidities that impact on 28-day mortality within the subgroups of patients with a specific infection; d) the influence of the number of comorbidities on 28-day mortality within the subgroups of patients with a specific infection; and e) if comorbidities as expressed by the CCI have a different impact for 28-day mortality in relation to the underlying type of infection.

### Statistical analysis

The Sepsis-3 expert panel has decided to introduce 90% sensitivity as the cut-off of discrimination in the analysis of Receiver Operator Characteristics (ROC) curves for variables that influence sepsis outcome [1]. As a consequence, we selected 90% sensitivity as the criterion to define a value of CCI that can discriminate an adequate probability for death after 28 days in the entire population. Specificity, positive and negative predictive value of the selected cut-off of CCI for 28-day mortality were also calculated. The odds ratio and 95% confidence intervals (CIs) for sepsis compared to non-sepsis at the selected CCI cut-off was calculated for patients with and without a specific type of infection; ORs were compared by the Tarone’s test. The same analysis of ORs was done for 28-day mortality. To define the role of each comorbidity, frequencies of each of the 18 comorbidities among non-sepsis and sepsis patients and among survivors and non-survivors were compared within each infection sub-group by the Fisher exact test. Comorbidities with a *p*-value of difference less than 0.05 entered a logistic forward conditional regression analysis to define the precise comorbidities that influence patients within each specific infection. The OR and 95%CIs for sepsis and for 28-day mortality in relation to the number of comorbidities was calculated; ORs were compared by the Tarone’s test. Any value of p below 0.05 was considered significant.

## Results

**Fig. 1:**
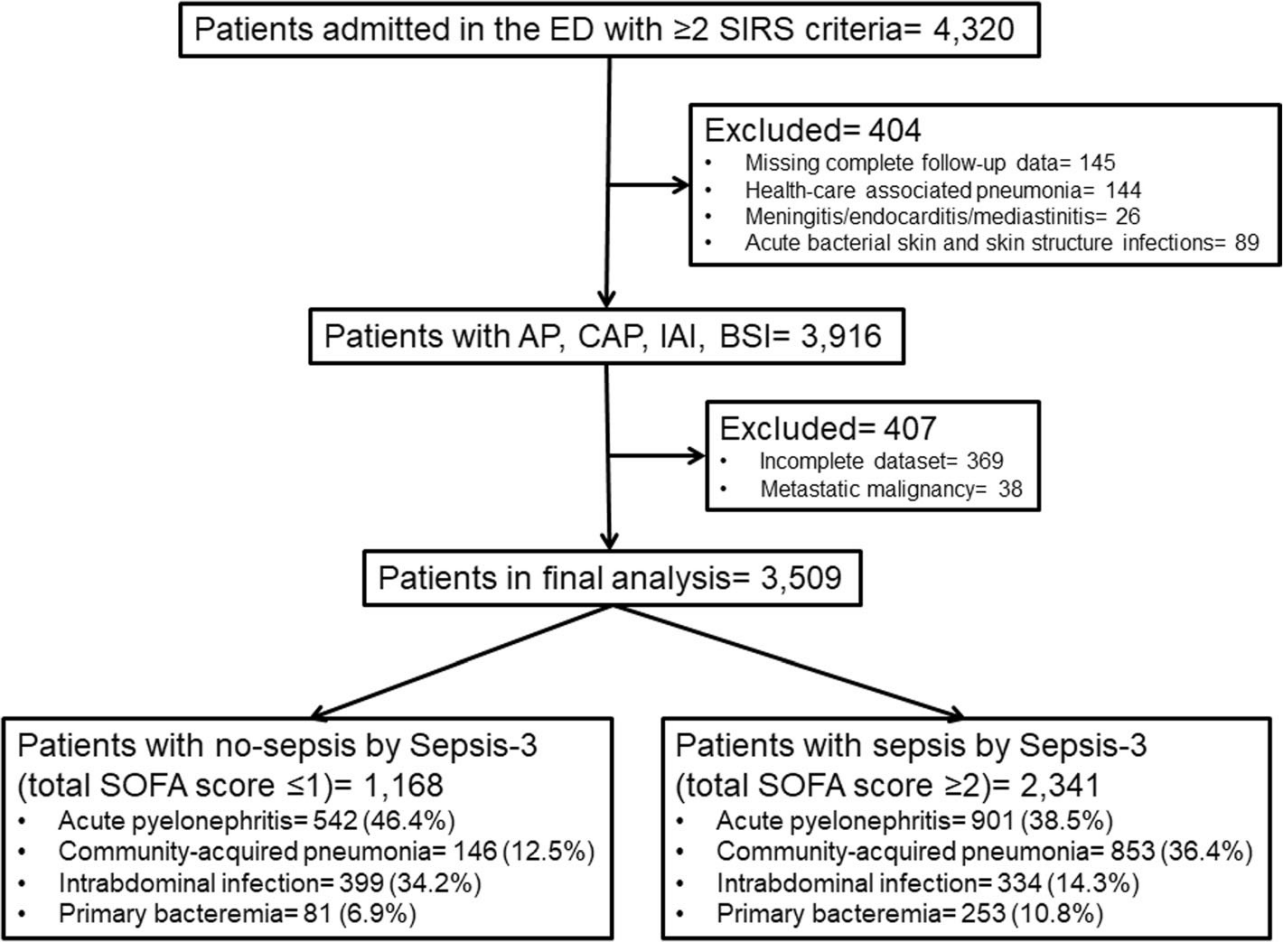
Study flow chart. AP: acute pyelonephritis; BSI: primary bacteremia; CAP: community-acquired pneumonia; ED: emergency department; IAI: intraabdominal infection; SIRS: systemic inflammatory response syndrome; SOFA: sequential organ failure assessment

The study flow chart is shown in Fig. 1. A total of 3509 patients were analyzed; 2341 had sepsis as defined by the new Sepsis-3 definitions. The baseline characteristics of these patients are shown in Table 1.

**Table 1:** Comparison of demographics of patients without sepsis and with sepsis in relation to the underlying infection

	Acute pyelonephritis			Community-acquired pneumonia			Intra-abdominal infections			Primary bacteremia		
	No sepsis	Sepsis	*p*	No sepsis	Sepsis	*p*	No sepsis	Sepsis	*p*	No sepsis	Sepsis	*p*
Number of patients	542	901		146	853		399	334		81	253	
Male gender (n, %)	145 (26.8)	361 (40.0)	< 0.0001	78 (53.4)	473 (55.4)	0.897	180 (45.2)	148 (45.1)	1.000	40 (49.4)	123 (48.6)	1.000
Age (mean ± SD, years)	61.1 ± 22.0	74.8 ± 15.3	< 0.0001	61.4 ± 20.2	75.3 ± 14.9	< 0.0001	55.4 ± 24.3	74.7 ± 14.8	< 0.0001	63.3 ± 19.7	72.9 ± 14.5	< 0.0001
APACHE II score (mean ± SD)	9.2 ± 6.0	16.5 ± 7.4	< 0.0001	8.4 ± 4.8	18.4 ± 7.6	< 0.0001	7.5 ± 4.9	17.5 ± 8.9	< 0.0001	9.2 ± 4.6	19.8 ± 7.5	< 0.0001
SOFA score (mean ± SD)	0.4 ± 0.5	5.9 ± 3.0	< 0.0001	0.7 ± 0.5	5.8 ± 3.2	< 0.0001	0.2 ± 0.5	5.3 ± 3.0	< 0.0001	0.5 ± 0.5	5.9 ± 3.4	< 0.0001
CCI (mean ± SD)	3.0 ± 2.5	5.0 ± 2.6	< 0.0001	2.7 ± 2.3	4.8 ± 2.4	< 0.0001	2.3 ± 2.3	4.6 ± 2.4	< 0.0001	3.0 ± 2.0	4.5 ± 2.4	< 0.0001
28-day mortality (n, %)	21 (3.9)	208 (23.1)	< 0.0001	7 (4.8)	323 (37.9)*	< 0.0001	17 (4.3)	109 (32.6)*	< 0.0001	4 (4.9)	98 (38.6)*	< 0.0001

Abbreviations: *APACHE* acute physiology and chronic health evaluation, *CCI* Charlson’s comorbidity index, *SOFA* sequential organ failure assessment

*p < 0.0001 compared to the respective mortality of acute pyelonephritis

### Primary study endpoint

**Fig. 2:**
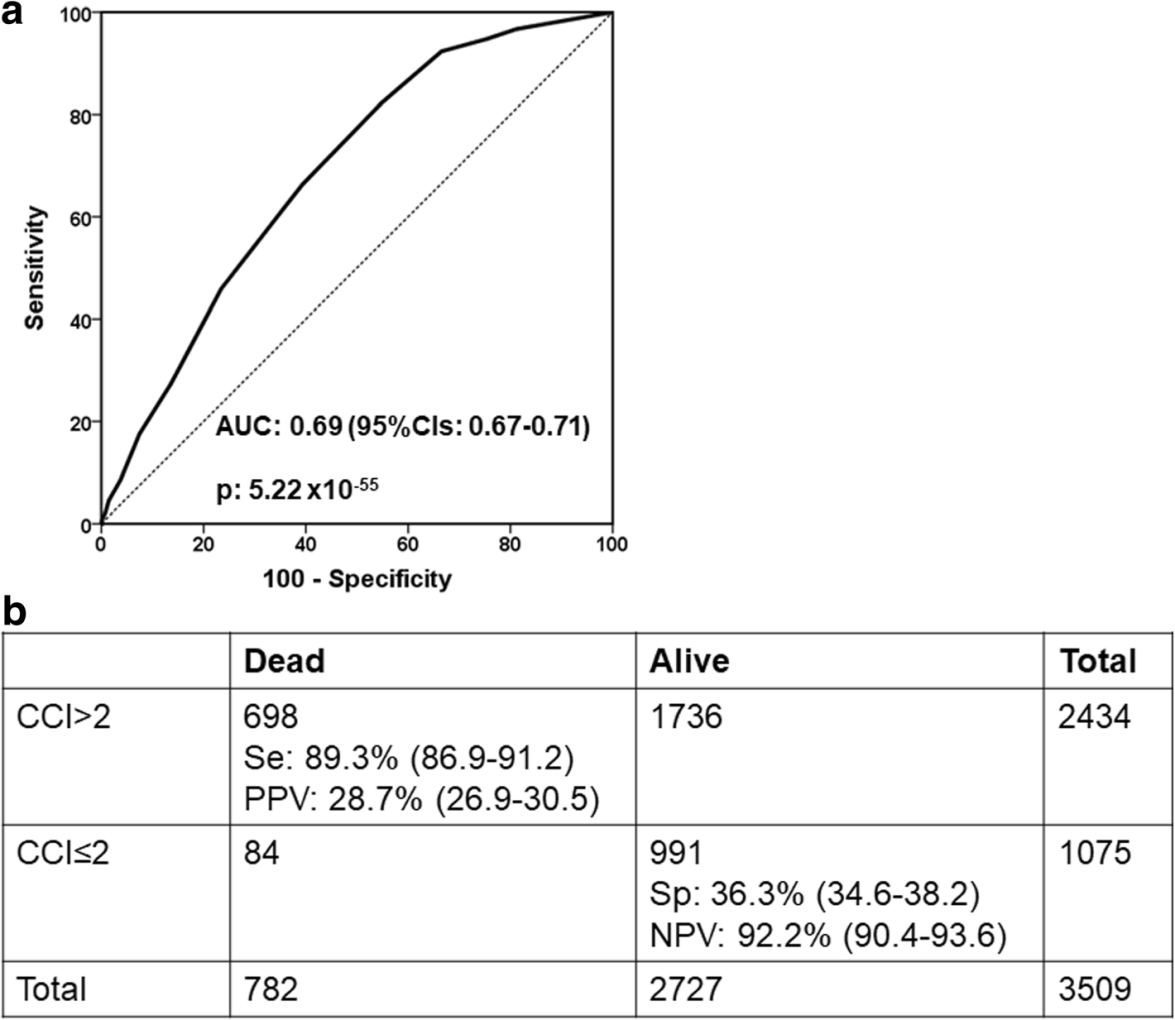
Charlson’s comorbidity index (CCI) influences final outcome. **a** ROC curve of CCI for 28-day mortality. **b** Prognostic performance of CCI more than 2 for 28-day mortality. NPV: negative predictive value; PPV: positive predictive value; Se: sensitivity; Sp: specificity

**Fig. 3:**
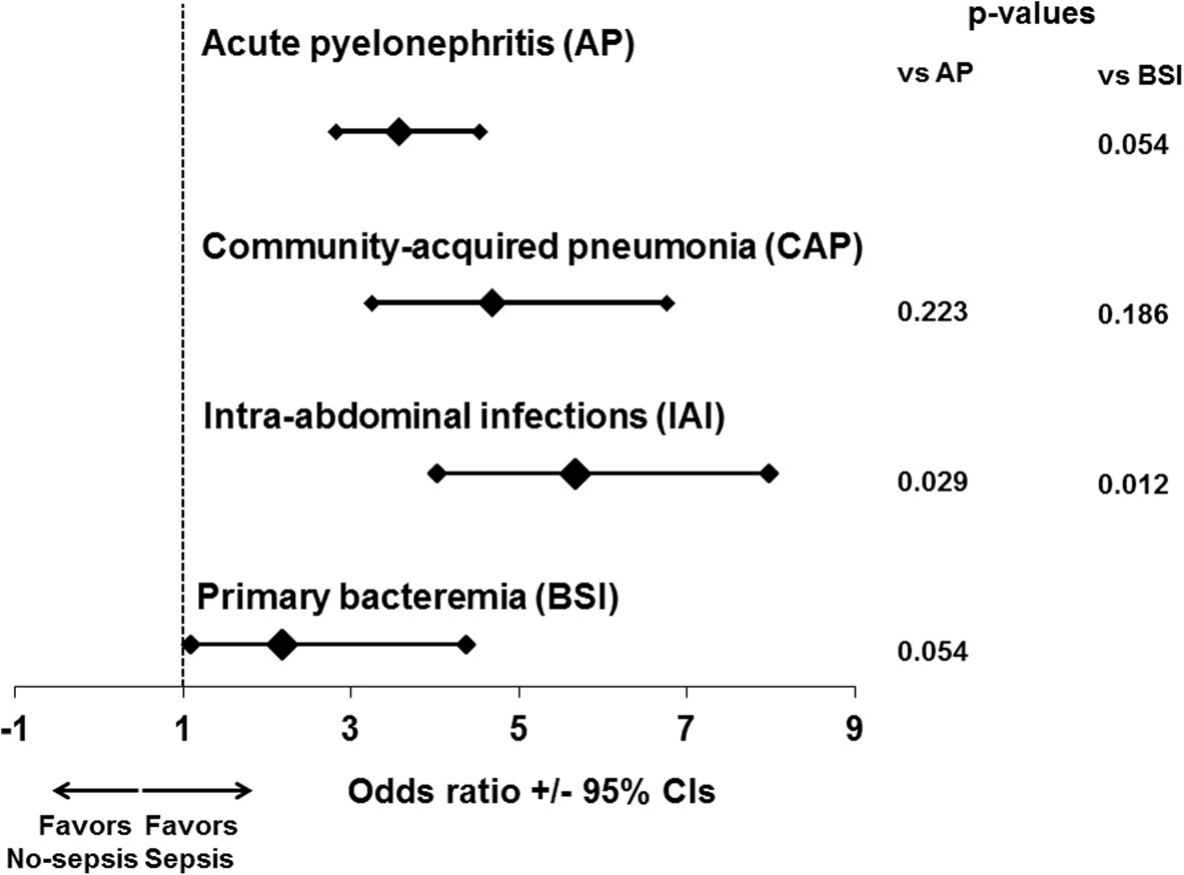
Modulation of the risk for sepsis in relation to the underlying infection and the Charlson’s comorbidity index (CCI). Each line represents the odds ratios and confidence intervals (CI) for death of each individual infection when CCI is more than 2 compared to CCI ≤2. AP: acute pyelonephritis; BSI: primary bacteremia; CAP: community-acquired pneumonia; IAI: intraabdominal infection

ROC curve analysis conducted in the overall study population showed that CCI more than 2 was accompanied by 89.3% sensitivity (86.9–91.2%) to predict 28-day mortality (Fig. 2). Figure 3 shows the ORs for the development of sepsis in relation to the underlying infection for patients with CCI more than 2. Findings suggest that although the OR for sepsis was significantly increased under the pressure of CCI more than 2 for all types of infection, this effect was far more pronounced for patients with IAIs.

### Secondary study endpoints

The next question was what the exact co-morbidities are that can enhance the development of sepsis among patients within each infection subgroup. At first, comparisons were done to define the comorbidities that differ between non-sepsis and sepsis patients within each infection subgroup. The analysis indicated 10 comorbidities that differ between non-sepsis and sepsis patients in the case of AP (Additional file 1: Table S1), seven comorbidities in the case of CAP (Additional file 2: Table S2), 11 comorbidities in the case of IAIs (Additional file 3: Table S3) and three comorbidities in the case of BSI (Additional file 4: Table S4). These comorbidities entered into a logistic forward conditional regression analysis to conclude which are the precise comorbidities that are associated with the development of sepsis within each subgroup. Seven comorbidities were found in the case of AP, four in the case of CAP, six in the case of IAIs and only one in the case of BSI (Table 2). Among patients with type 2 diabetes mellitus, the need for intake of insulin for glycemic control did not modify the risk for sepsis compared to diabetic patients without insulin intake in the case of AP (OR: 1.29; 95%CIs: 0.86–1.95; *p*: 0.212), of CAP (OR: 1.15; 95%CIs: 0.46–2.87; p: 0.761) and of IAIs (OR: 1.84; 95%CIs: 0.87–3.90; p: 0.113). Among patients with chronic heart failure, those at end stage had greater risk for sepsis in the case of AP (OR: 5.40; 95%CIs: 2.22–13.14; *p* < 0.0001) but not of CAP (OR: 1.73; 95%CIs: 0.54–5.56; *p*: 0.357). Among patients with chronic renal disease, the number of patients who developed sepsis and who were on chronic hemodialysis was too low to allow stratification by disease severity.

**Table 2:** Impact of precise co-morbidities on the development of sepsis

Co-morbidity	Odds ratio	95% confidence intervals	*p*-value
Patients with acute pyelonephritis			
Type 2 diabetes mellitus	1.31	1.02–1.68	0.033
Chronic heart failure	1.93	1.39–2.69	< 0.0001
Chronic renal disease	29.31	9.26–92.86	< 0.0001
Non-metastatic solid tumor malignancy	2.03	1.40–2.89	< 0.0001
Corticosteroid intake	2.08	1.08–3.98	0.028
Stroke	1.70	1.21–2.39	0.002
Dementia	1.97	1.37–2.84	< 0.0001
Patients with community-acquired pneumonia			
Type 2 diabetes mellitus	1.73	1.06–2.82	0.027
Chronic heart failure	1.99	1.13–3.49	0.016
Coronary heart disease	3.72	1.69–8.19	0.001
Dementia	3.44	1.64–7.24	0.001
Patients with intraabdominal infections			
Type 2 diabetes mellitus	3.29	2.15–5.03	< 0.0001
Chronic renal disease	26.77	3.51–204.37	0.002
Corticosteroid intake	3.86	1.41–10.53	0.008
Atrial fibrillation	2.73	1.41–5.28	0.003
Dementia	9.33	3.79–22.97	< 0.0001
Liver cirrhosis	9.16	1.06–79.53	0.044
Patients with primary bacteremia			
Dementia	8.55	1.12–65.20	0.038

Only variables remaining significant after the final step of logistic forward conditional regression analysis are included

**Fig. 4:**
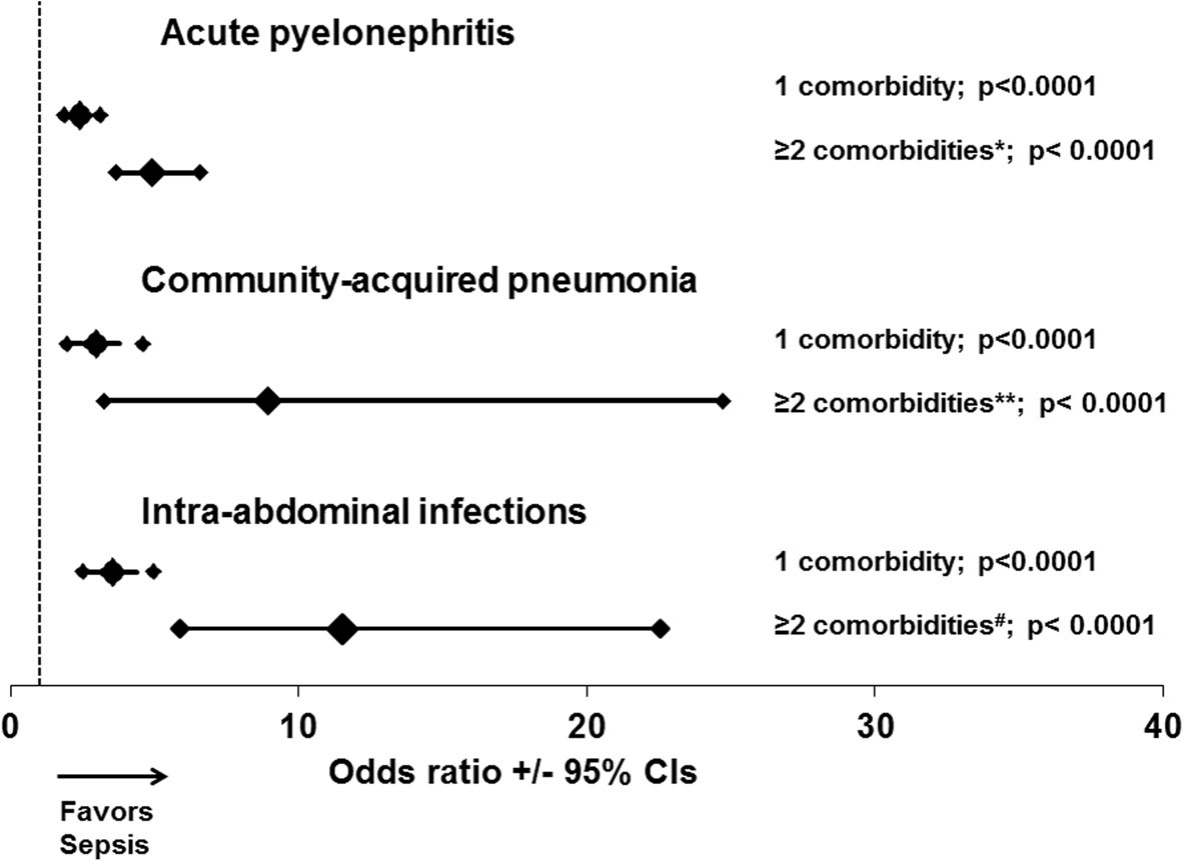
Modulation of the risk for sepsis in relation to the underlying infection and the number of comorbidities. Each line represents the odds ratios and confidence intervals (CI) for sepsis in the presence of one or at least two comorbidities, as defined for each infection in Table 1. *P* values represent comparisons with patients without any comorbidity. The *p*-values of comparisons between odds ratio for one comoborditiy and for at least two comorbidities are: *0.00002; **0.033; ^#^0.0018

One major question was whether the number of comorbidities may influence the susceptibility for sepsis. Figure 4 shows the ORs for sepsis for each individual infection under the pressure of one or at least two comorbidities compared to the absence of comorbidities. Even the presence of at least one of the comorbidities listed in Table 2 increased significantly the risk for sepsis. In all types of infection, the OR under the pressure of two or more comorbidities was significantly greater than under the pressure of only one comorbidity.

The comorbidities found in Table 2 to impose considerably for the development of sepsis entered into a logistic forward conditional regression analysis to decipher their impact on 28-day mortality within infection subgroups. Six comorbidities were found in the case of AP, two in the case of CAP, six in the case of IAIs and only one in the case of BSI (Table 3). Among patients with chronic heart failure, those at end stage had greater risk for sepsis in the case of AP (OR: 1.18; 95%CIs: 0.68–2.05; p: 0.543). Among patients with type 2 diabetes mellitus, the need for intake of insulin for glycemic control did not modify the risk for death in the case of IAIs (OR: 1.21; 95%CIs: 0.58–2.51; p: 0.607).

**Table 3:** Impact of precise co-morbidities on 28-day mortality

Co-morbidity	Odds ratio	95% confidence intervals	*p*-value
Patients with acute pyelonephritis			
Chronic heart failure	2.54	1.82–3.55	< 0.0001
Chronic renal disease	1.71	1.13–2.60	0.011
Non-metastatic solid tumor malignancy	2.13	1.44–3.17	< 0.0001
Corticosteroid intake	2.05	1.09–3.84	0.024
Stroke	2.97	2.12–4.17	< 0.0001
Dementia	2.18	1.51–3.15	< 0.0001
Patients with community-acquired pneumonia			
Coronary heart disease	1.87	1.30–2.69	0.001
Dementia	2.20	1.53–3.17	< 0.0001
Patients with intraabdominal infections			
Type 2 diabetes mellitus	1.84	1.16–2.93	0.010
Chronic renal disease	2.67	1.12–6.35	0.026
Non-metastatic solid tumor malignancy	3.03	1.79–5.13	< 0.0001
Atrial fibrillation	2.23	1.11–4.48	0.024
Dementia	3.69	1.87–7.25	< 0.0001
Liver cirrhosis	4.59	1.20–17.51	0.025
Patients with primary bacteremia			
Dementia	3.87	1.65–9.12	0.002

Only variables remaining significant after the final step of logistic forward conditional regression analysis are included

**Fig. 5:**
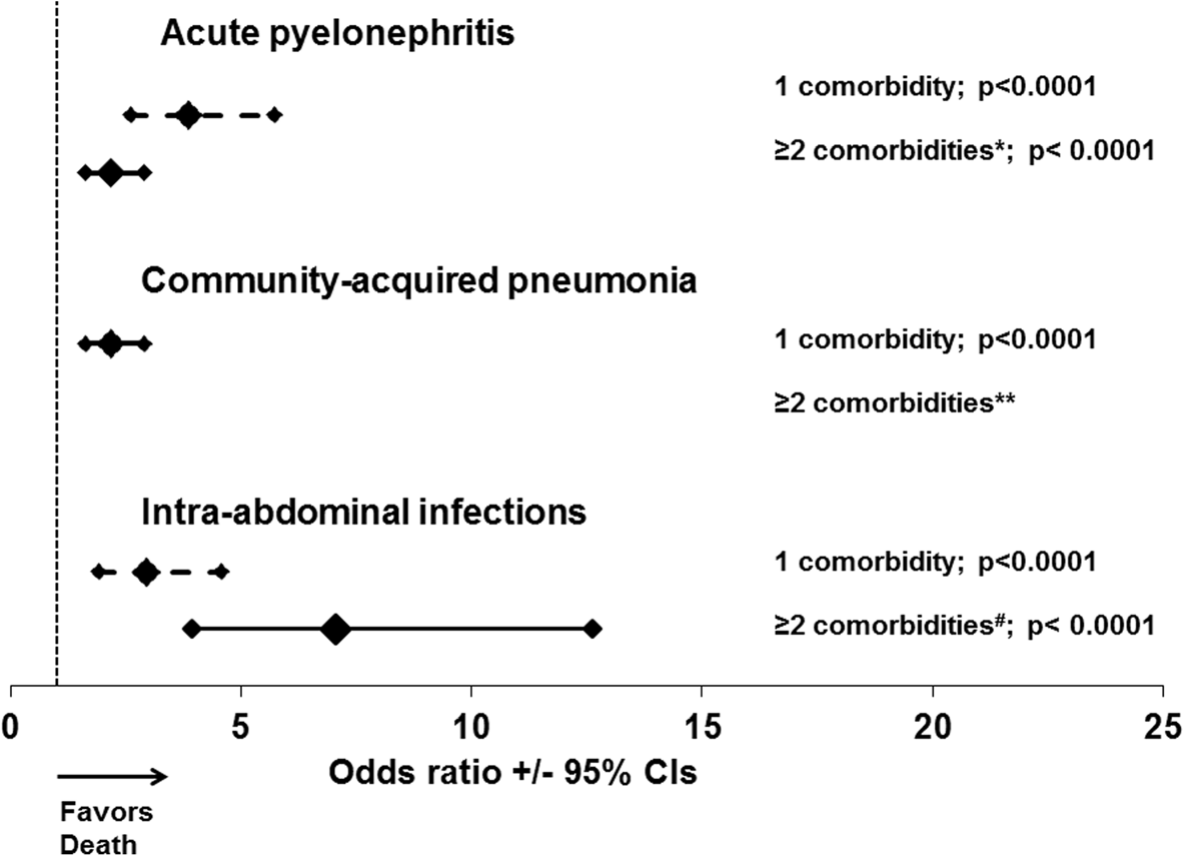
Modulation of the risk for death after 28 days in relation to the underlying infection and the number of comorbidities. Each line represents the odds ratios and confidence intervals (CI) for death in the presence of one or at least two comorbidities, as defined for each infection in Table 2. *P* values represent comparisons with patients without any comorbidity. The *p*-values of comparisons between odds ratio for one moborditiy and for at least two comorbidities are: *0.00002; ^#^0.0029. **could not be calculated because one value was zero

Figure 5 shows the odds ratios for death by each infection under the pressure of one or at least two comorbidities compared to the absence of comorbidities. Even the presence of at least one of the comorbidities listed in Table 3 significantly increased the risk for death. In all types of infection, the OR for death under the pressure of two or more comorbidities was significantly greater than under the pressure of only one comorbidity.

**Fig. 6:**
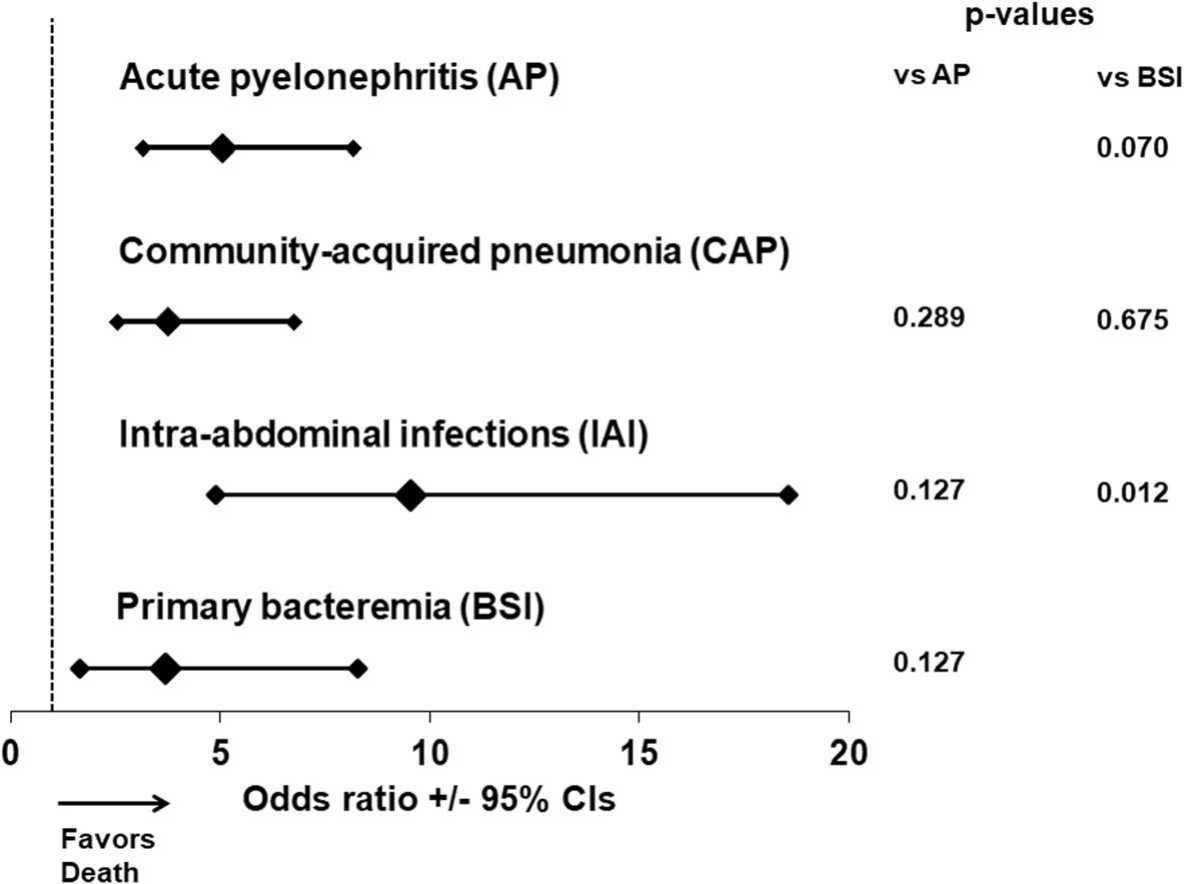
Modulation of the risk for 28-day mortality in relation to the underlying infection and the Charlson’s comorbidity index (CCI). Each line represents the odds ratios and confidence intervals (CI) for death of each individual infection when CCI is more than 2 compared to CCI ≤2. *P*-values are compared by the Tarone’s test. AP: acute pyelonephritis; BSI: primary bacteremia; CAP: community-acquired pneumonia; IAI: intraabdominal infection

Although the OR for 28-day mortality was significantly increased under the pressure of CCI more than 2 for all types of infection, this effect was far more pronounced for patients with IAIs (Fig. 6).

## Discussion

The present analysis managed to demonstrate that the risk for the development of sepsis, as this is defined by the new Sepsis-3 definitions, is modified in an individualized way in relation to the type of underlying infection. Findings clearly showed that comorbidities increased considerably the risk for sepsis and for unfavorable outcome after 28-days and that this effect varied greatly with the number of existing comorbidities. When using CCI as an expression of the constellation of comorbidities of the host, it was found that the susceptibility for both the development of sepsis and death after 28 days was far greater under the pressure of CCI more than 2 in intraabdominal infections that with any other type of infection. Regarding the influence of individual comorbidities, some comorbidities like type 2 diabetes mellitus, chronic renal disease and dementia were associated with sepsis risk in almost all types of infection. Others like chronic heart disease and non-metastatic tumor malignancy introduced sepsis risk in CAP and IAI, whereas coronary heart disease introduced risk only in CAP. Atrial fibrillation and liver cirrhosis increased sepsis risk only in IAI. Surprisingly, the risk for sepsis after BSI was increased only in demented patients. A similar pattern was found regarding the impact of each comorbidity on 28-day mortality. It should be underscored that risk for sepsis and death was not significantly modified among patients with advanced type 2 diabetes mellitus and chronic heart failure compared to the less advanced disease state.

There is a great difference between susceptibility to an infection and susceptibility to inflammation. Many studies have shown the role of type 2 diabetes mellitus, solid tumor and hematologic malignancies, liver cirrhosis, atrial fibrillation and coronary heart disease for susceptibility to infections [6–10]. In this study, we try to define which comorbidities elicit progression to organ dysfunction once an infection has started. Of course this cannot be done with comparison of infected patients with healthy controls. Instead we compared various non-serious with serious community-acquired infections admitted to the ED. Our findings agree at some aspects and disagree at some other aspects with the current ideas about comorbidities that define risk for severity. A typical example is the case of CAP. Severity of CAP is defined by the pneumonia severity index (PSI) in which the history of five disorders i.e. neoplastic disease, congestive heart failure, cerebrovascular disease, renal disease and liver disease are taken into consideration [11]. Our analysis shows that among these five comorbidities only chronic heart disease leads the development of organ dysfunction.

The impact of diabetes mellitus type 2 on the final outcome of patients with sepsis is a matter of debate. A comparison of the mortality of 241 diabetic patients and 863 non-diabetic patients with sepsis was done in the prospective cohort of the Molecular Diagnosis and Risk Stratification of Sepsis (MARS) project of two large academic centers in the Netherlands [12]. No difference in both short- and long-term outcomes was found and this was accompanied with lack of differences in the levels of circulating biomarkers for inflammation, coagulation and endothelial activation. The same lack of effect on clinical outcomes and concentrations of biomarkers was found after adjustment for treatment with insulin and metformin [12]. This finding corroborates our results on the lack of effect of type 2 diabetes mellitus as a risk factor for 28-day mortality in AP and CAP. The impact of type 2 diabetes on the final outcome of CAP was also studied in two big cohorts, the GenIMS of 1895 subjects with CAP and the Health ABC of 1645 subjects. Mortality was greater among patients with diabetes than without diabetes [13]. At first reading, this finding is opposite to the lack of association between type 2 diabetes and mortality from CAP described in our study. However, diabetic patients of the GenIMS and Health ABC cohorts had greater risk for death by cardiovascular events [13]. This is partly compatible with our finding for coronary heart disease as an independent risk factor for death in CAP.

Sepsis is a multifactorial process and staging is necessary to provide personalized treatment targeting the needs of each patient. This concept has been introduced may years ago where the PIRO system was introduced. The acronym of PIRO stands for predisposition through comorbidities, infection, response of the host and organ dysfunction [6]. Our analysis showed for the first time an additive interaction between comorbidities and IAIs that increased the likelihood for sepsis and unfavorable outcome far more than the other types of infection. The data make clear that the PIRO system should separately stage the significance of the six comorbidities affecting outcome in IAI.

## Conclusion

The results of our study generate the need to consider development of sepsis and organ dysfunction after an infection an individualized process. Comorbidities play a major role in this process. However, the comorbidities which facilitate progression into organ dysfunction vary according to the underlying infection. Among all type of infections, IAIs act additively with the comorbidities of the host to potentiate the likelihood for sepsis and the risk for unfavorable outcome at an extent much greater than the other infections.

## Additional files

Additional file 1: **Table S1.** Comparison of comorbidities between patients with infection and sepsis developing in the field of acute pyelonephritis. (DOCX 21 kb)

Additional file 2: **Table S2.** Comparison of comorbidities between patients with infection and sepsis developing in the field of community-acquired pneumonia. (DOCX 21 kb)

Additional file 3: **Table S3.** Comparison of comorbidities between patients with infection and sepsis developing in the field of intraabdominal infections. (DOCX 20 kb)

Additional file 4: **Table S4.** Comparison of comorbidities between patients with infection and sepsis developing in the field of primary bacteremia. (DOCX 21 kb)

## Abbreviations

AP: Acute pyelonephritis
APACHE: Acute physiology and chronic health evaluation
BSI: Primary bacteremia
CAP: Community-acquired pneumonia
CCI: Charlson’s co-morbidity index
COPD: Chronic obstructive pulmonary disorder
ED: Emergency department
HSSG: Hellenic sepsis study group
IAI: Intraabdominal infections
MARS: Molecular Diagnosis and Risk Stratification of Sepsis
PSI: Pneumonia severity index
ROC: Receiver Operator Characteristics
SIRS: Systemic inflammatory response syndrome
SOFA: Sequential organ failure assessment
