# Supplementary material for: Progression into sepsis: an individualized process varying by the interaction of comorbidities with the underlying infection
Source: BMC Infect Dis. 2018 May 29;18:242. doi: 10.1186/s12879-018-3156-z (PMC5975439; doi:10.1186/s12879-018-3156-z)
Supplement: Supplementary file 1 — Table S1. Comparison of comorbidities between patients with infection and sepsis developing in the field of acute pyelonephritis. (DOCX 21 kb) [file 12879_2018_3156_MOESM1_ESM.docx]

**Additional Table 1** Comparison of comorbidities between patients with infection and sepsis developing in the field of acute pyelonephritis.

| **Co-morbidity (n, %)** | **No sepsis (n= 542)** | **Sepsis (n= 901)** | **p-value** |
| --- | --- | --- | --- |
| Type 2 diabetes mellitus | 145 (26.8) | 326 (36.2) | <0.0001 |
| Chronic heart failure | 59 (10.9) | 211 (23.4) | <0.0001 |
| Chronic obstructive pulmonary disease | 31 (5.7) | 91 (10.1) | 0.003 |
| Chronic renal disease | 3 (0.6) | 147 (16.3) | <0.0001 |
| Non-metastatic solid tumor malignancy | 49 (9.1) | 144 (16.0) | <0.0001 |
| Corticosteroid intake | 13 (2.4) | 48 (5.3) | 0.007 |
| Coronary heart disease | 28 (5.2) | 89 (9.9) | 0.001 |
| Vascular hypertension | 79 (14.6) | 135 (15.0) | 0.879 |
| Atrial fibrillation | 32 (5.9) | 91 (10.1) | 0.006 |
| Dyslipidemia | 34 (6.3) | 65 (7.2) | 0.521 |
| Stroke | 58 (10.8) | 189 (21.0) | <0.0001 |
| Dementia | 47 (8.7) | 158 (17.5) | <0.0001 |
| Nephrolithiasis | 135 (25.0) | 223 (24.8) | 0.900 |
| Gallstones | 28 (5.2) | 63 (7.0) | 0.181 |
| Liver cirrhosis | 1 (0.2) | 9 (1.0) | 0.101 |
| Obesity | 16 (3.0) | 28 (3.1) | 1.000 |
| Depression | 4 (0.7) | 9 (1.0) | 0.777 |
