# Supplementary material for: Progression into sepsis: an individualized process varying by the interaction of comorbidities with the underlying infection
Source: BMC Infect Dis. 2018 May 29;18:242. doi: 10.1186/s12879-018-3156-z (PMC5975439; doi:10.1186/s12879-018-3156-z)
Supplement: Supplementary file 2 — Table S2. Comparison of comorbidities between patients with infection and sepsis developing in the field of community-acquired pneumonia. (DOCX 21 kb) [file 12879_2018_3156_MOESM2_ESM.docx]

**Additional Table 2** Comparison of comorbidities between patients with infection and sepsis developing in the field of community-acquired pneumonia.

| **Co-morbidity (n, %)** | **No sepsis (n= 146)** | **Sepsis (n= 853)** | **p-value** |
| --- | --- | --- | --- |
| Type 2 diabetes mellitus | 23 (15.8) | 254 (29.8) | <0.0001 |
| Chronic heart failure | 16 (11.0) | 207 (24.3) | <0.0001 |
| Chronic obstructive pulmonary disease | 18 (12.3) | 159 (18.6) | 0.078 |
| Chronic renal disease | 0 (0) | 60 (7.1) | <0.0001 |
| Non-metastatic solid tumor malignancy | 13 (9.0) | 119 (14.0) | 0.112 |
| Corticosteroid intake | 5 (3.4) | 50 (5.9) | 0.325 |
| Coronary heart disease | 7 (4.8) | 139 (16.3) | <0.0001 |
| Vascular hypertension | 17 (11.6) | 173 (20.3) | 0.012 |
| Atrial fibrillation | 7 (4.8) | 131 (15.4) | <0.0001 |
| Dyslipidemia | 10 (6.8) | 104 (12.2) | 0.068 |
| Stroke | 13 (8.9) | 187 (22.1) | <0.0001 |
| Dementia | 8 (5.5) | 135 (15.9) | <0.0001 |
| Nephrolithiasis | 2 (1.4) | 19 (2.2) | 0.756 |
| Gallstones | 6 (4.1) | 44 (5.2) | 0.686 |
| Liver cirrhosis | 0 (0) | 5 (0.6) | 1.000 |
| Obesity | 6 (4.1) | 15 (1.8) | 0.108 |
| Depression | 0 (0) | 13 (1.5) | 0.235 |
