# Supplementary material for: Progression into sepsis: an individualized process varying by the interaction of comorbidities with the underlying infection
Source: BMC Infect Dis. 2018 May 29;18:242. doi: 10.1186/s12879-018-3156-z (PMC5975439; doi:10.1186/s12879-018-3156-z)
Supplement: Supplementary file 3 — Table S3. Comparison of comorbidities between patients with infection and sepsis developing in the field of intraabdominal infections. (DOCX 20 kb) [file 12879_2018_3156_MOESM3_ESM.docx]

**Additional Table 3** Comparison of comorbidities between patients with infection and sepsis developing in the field of intraabdominal infections.

| **Co-morbidity (n, %)** | **No sepsis (n= 399)** | **Sepsis (n= 334)** | **p-value** |
| --- | --- | --- | --- |
| Type 2 diabetes mellitus | 42 (10.5) | 101 (30.2) | <0.0001 |
| Chronic heart failure | 48 (12.0) | 70 (21.0) | 0.001 |
| Chronic obstructive pulmonary disease | 28 (7.0) | 33 (9.9) | 0.180 |
| Chronic renal disease | 1 (0.3) | 26 (7.8) | <0.0001 |
| Non-metastatic solid tumor malignancy | 28 (7.0) | 56 (16.8) | <0.0001 |
| Corticosteroid intake | 6 (1.5) | 17 (5.1) | 0.009 |
| Coronary heart disease | 19 (4.8) | 34 (10.2) | 0.006 |
| Vascular hypertension | 44 (11.0) | 63 (18.9) | 0.003 |
| Atrial fibrillation | 17 (4.3) | 31 (9.3) | 0.007 |
| Dyslipidemia | 15 (3.8) | 14 (4.2) | 0.850 |
| Stroke | 23 (5.8) | 42 (12.6) | 0.002 |
| Dementia | 6 (1.5) | 38 (11.4) | <0.0001 |
| Nephrolithiasis | 7 (1.8) | 11 (3.3) | 0.232 |
| Gallstones | 111 (27.8) | 102 (30.5) | 0.462 |
| Liver cirrhosis | 1 (0.3) | 9 (2.7) | 0.007 |
| Obesity | 7 (1.8) | 13 (3.9) | 0.109 |
| Depression | 1 (0.3) | 2 (0.6) | 0.593 |
