# Supplementary material for: Progression into sepsis: an individualized process varying by the interaction of comorbidities with the underlying infection
Source: BMC Infect Dis. 2018 May 29;18:242. doi: 10.1186/s12879-018-3156-z (PMC5975439; doi:10.1186/s12879-018-3156-z)
Supplement: Supplementary file 4 — Table S4. Comparison of comorbidities between patients with infection and sepsis developing in the field of primary bacteremia. (DOCX 21 kb) [file 12879_2018_3156_MOESM4_ESM.docx]

**Additional Table 4** Comparison of comorbidities between patients with infection and sepsis developing in the field of primary bacteremia.

| **Co-morbidity (n, %)** | **No sepsis (n= 81)** | **Sepsis (n= 253)** | **p-value** |
| --- | --- | --- | --- |
| Type 2 diabetes mellitus | 9 (11.1) | 58 (22.9) | 0.149 |
| Chronic heart failure | 3 (3.7) | 39 (15.4) | 0.030 |
| Chronic obstructive pulmonary disease | 4 (4.9) | 17 (6.7) | 1.000 |
| Chronic renal disease | 0 (0) | 43 (16.9) | <0.0001 |
| Non-metastatic solid tumor malignancy | 8 (9.9) | 30 (11.9) | 0.825 |
| Corticosteroid intake | 1 (1.2) | 11 (4.4) | 0.469 |
| Coronary heart disease | 2 (2.5) | 20 (7.9) | 0.264 |
| Vascular hypertension | 9 (11.1) | 28 (11.1) | 0.498 |
| Atrial fibrillation | 1 (1.2) | 23 (9.1) | 0.055 |
| Dyslipidemia | 3 (3.7) | 15 (5.9) | 1.000 |
| Stroke | 6 (7.4) | 31 (12.3) | 0.103 |
| Dementia | 1 (1.2) | 24 (9.5) | 0.034 |
| Nephrolithiasis | 1 (1.2) | 8 (3.2) | 1.000 |
| Gallstones | 2 (2.5) | 12 (4.74) | 1.000 |
| Liver cirrhosis | 1 (1.2) | 2 (0..8) | 0.485 |
| Obesity | 1 (1.2) | 8 (3.2) | 1.000 |
| Depression | 2 (2.5) | 2 (0.8) | 0.486 |
